# Cross-species comparisons reveal resistance of human skeletal stem cells to inhibition by non-steroidal anti-inflammatory drugs

**DOI:** 10.3389/fendo.2022.924927

**Published:** 2022-08-25

**Authors:** L. Henry Goodnough, Thomas H. Ambrosi, Holly M. Steininger, M. Gohazrua K. Butler, Malachia Y. Hoover, HyeRan Choo, Noelle L. Van Rysselberghe, Michael J. Bellino, Julius A. Bishop, Michael J. Gardner, Charles K. F. Chan

**Affiliations:** ^1^ Department of Orthopaedic Surgery, Stanford Hospitals and Clinics, Stanford, CA, United States; ^2^ Institute for Stem Cell Biology and Regenerative Medicine, Stanford University School of Medicine, Stanford, CA, United States; ^3^ Division of Plastic and Reconstructive Surgery, Department of Surgery, Stanford University School of Medicine, Stanford, CA, United States

**Keywords:** skeletal stem cells (SSCs), non-steroid antiinflamatory drugs, species specificity, bone regeneration, inflammation, fracture healing

## Abstract

Fracture healing is highly dependent on an early inflammatory response in which prostaglandin production by cyclo-oxygenases (COX) plays a crucial role. Current patient analgesia regimens favor opioids over Non-Steroidal Anti-Inflammatory Drugs (NSAIDs) since the latter have been implicated in delayed fracture healing. While animal studies broadly support a deleterious role of NSAID treatment to bone-regenerative processes, data for human fracture healing remains contradictory. In this study, we prospectively isolated mouse and human skeletal stem cells (SSCs) from fractures and compared the effect of various NSAIDs on their function. We found that osteochondrogenic differentiation of COX2-expressing mouse SSCs was impaired by NSAID treatment. In contrast, human SSCs (hSSC) downregulated COX2 expression during differentiation and showed impaired osteogenic capacity if COX2 was lentivirally overexpressed. Accordingly, short- and long-term treatment of hSSCs with non-selective and selective COX2 inhibitors did not affect colony forming ability, chondrogenic, and osteogenic differentiation potential *in vitro*. When hSSCs were transplanted ectopically into NSG mice treated with Indomethacin, graft mineralization was unaltered compared to vehicle injected mice. Thus, our results might contribute to understanding species-specific differences in NSAID sensitivity during fracture healing and support emerging clinical data which conflicts with other earlier observations that NSAID administration for post-operative analgesia for treatment of bone fractures are unsafe for patients.

## Introduction

Non-steroidal anti-inflammatory drugs (NSAIDs) are commonly used for pain relief after operative treatment of fractures, and there is significant clinical interest as to whether NSAID administration itself has a deleterious effect on fracture healing in humans. NSAIDs inhibit cyclo-oxygenase (COX) enzymes, including COX1 and COX2, that mitigate prostaglandin production and pain. Whether NSAIDs affect osteoblast progenitor differentiation, inhibit fracture healing, and therefore increase the risk of nonunion, remains controversial. Experimental rodent models overwhelmingly have suggested that NSAID administration inhibits new bone formation and fracture healing (1–4). However, the corresponding generalizability of findings in murine models to humans is uncertain. In humans, the role of NSAIDs on fracture healing is inconclusive. In the clinical setting, historical retrospective studies suggested an association between NSAID use and fracture nonunion, but overall there is a lack of high quality, prospective evidence to conclusively demonstrate a relationship between NSAIDs and delayed union or nonunion (5–8). Mixed data from isolated skeletal cell populations tested for differentiation potential when treated with NSAID has contributed to this dilemma (9, 10). One reason explaining this dichotomy is the fact that bone marrow stromal cells are isolated retrospectively by plastic adherence which yield heterogeneous cell populations thereby leading to varying results (11, 12). We recently demonstrated that mouse and human osteochondrogenic cell types arise from a defined skeletal stem cell (SSC), a self-renewing, multi-potential population giving rise to a transient bone-cartilage-stromal-progenitor (BCSP) that can be isolated by fluorescence-activated cell sorting (FACS) from acute fractures based on their differential expression of a combination of specific cell surface markers. These cells show age-related functional impairments and might also be useful for prospectively assaying fracture healing outcome (13–17). Here, we reasoned that if we compared the effect of NSAIDs on freshly purified, functionally defined skeletal lineage cell types in mice and humans, we might be able to delineate species-specific effects on their response regarding bone-forming characteristics.

## Materials and methods

### Study approval

Studies involving the sourcing of human samples was approved by the Stanford IRB. Animal experiments complied with all relevant ethical regulations and were conducted under approved protocols by Stanford’s Administrative Panel on Laboratory Animal Care.

### Human tissue

hSSCs, human osteoprogenitors (hOPs), and human chondroprogenitors (hCPs) were collected from acute human fractures, and collected for transcriptomic analysis or expanded in culture medium, and differentiated in the presence or absence of NSAIDs or selective COX2 inhibitors as described before (13). Tissues were collected from acute fractures undergoing direct reduction and fixation. As per our previous observation that hSSCs from different long bone fracture sites are functionally identical, we have included specimens from tibial, humerus, radius and ulna fractures of patients aged 18 to 74 years (16). Any soft callus hematoma, which was impeding fracture reduction and considered medical waste, was collected.

### Mouse experiments

All animal experiments complied with all relevant ethical regulations and were conducted under approved protocols by Stanford’s Administrative Panel on Laboratory Animal Care. Mice were maintained at the Stanford University Research Animal Facility in accordance with Stanford University guidelines. Animals were given food and water ad libitum and housed in temperature-, moisture-, and light-controlled (12h light/dark cycle) micro-insulators. Fracture experiments were conducted on adult (10-12 weeks) male C57BL6/J mice. Subcutaneous transplants of human SSCs were performed in adult male NSG mice (NOD scid gamma; JAX: 005557).

### Skeletal stem cell isolation

hSSC were collected as previously described (16). Briefly, the tissue was initially minced with razor blades, collected in 0.22% collagenase digestion buffer (Sigma-Aldrich, Cat#C6885), and incubated at 37°C for 60 minutes under constant agitation. The supernatant was collected and filtered through a 70 µm nylon mesh and quenched in staining media (2% fetal calf serum, FCS, in phosphate-buffered saline, PBS) for subsequent centrifugation at 200 x g at 4°C and resuspension in staining media. Human skeletal cells were separated from RBCs by ACK lysis and washed with staining media. Cells were stained with fluorochrome-conjugated antibodies against CD45, CD235, CD31, TIE2, CD146, Podoplanin, CD164, CD73 (1:50; eBioscience). Flow cytometry was performed on a FACS Aria II. Gating schemes were established with fluorescence-minus-one (FMO) isotype controls and DAPI was used for viability staining. Human SSCs were isolated by CD45-CD235-CD31-TIE2-CD146-PDPN+CD164+CD73+ and hOPs by CD45-CD235-CD31-TIE2-CD146+PDPN- selection gated from single living cells (DAPI-negative).

Mouse SSCs were isolated from 10-day old femoral fracture calluses. Stabilized mid-diaphyseal femoral bi-cortical fractures were generated after inserting an intramedullary pin. Soft tissue-free femurs were processed as described before (18), antibody stained for CD45, Ter119, Thy1.1, Thy1.2, CD105, CD51, 6c3, Tie2, CD200 (eBiosciences) prior to isolation by flow cytometry. Mouse SSCs were isolated by CD45-Ter119-Thy1-CD105-CD51+6c3-Tie2-CD200+ selection gated from single living cells (Propidium Iodide-negative).

### Tissue culture and *in vitro* differentiation

For mouse and human colony forming assays, cells were plated at clonal density (defined number of 100 to 500 cells per well of a 6-well plate depending on experiment) and cultured in MEM alpha medium with 10% FBS and 1% pen strep (mouse) or 10% HPL, 1% pen strep, 0.01% heparin (human) maintained at 37°C incubator with 5% CO_2_. After two weeks cells were fixed, stained with 0.5% Crystal Violet and examined under phase microscopy and counted.

Osteogenic differentiation media (ODM) (MEM alpha medium, 10% Fetal Bovine Serum, 1% pen strep, 100 nM dexamethasone, 10 mM sodium β-glycerophosphate, 2.5 mM ascorbic acid) was changed every 3 days for 14 days. Cells were then stained with Alizarin Red to assess osteogenic potential. Alizarin red staining was quantified using spectrophotometry. Chondrogenesis assays were conducted in micromasses. Briefly, cells were resuspended at a cell-density of 1.6x10^7^ cells/ml. A 5 µl droplet of the cell suspension was seeded under high humidity conditions in a 24-well plate for 2 hr. After 2 hr, warmed chondrogenic differentiation media was added to the culture vessel. The growing micromass was fed with fresh chondrogenesis media (DMEMhigh [Thermo Fisher Scientific, Cat# 10569010] with 10% FBS, 100 nM dexamethasone, 1 µM ascorbic acid 2-phosphate, and 10 ng/ml TGFβ1 [Peprotech, Cat# 100-21C]) every other day in a 37°C incubator with 5% CO_2_. At day 14 the micromass was fixed and stained with Alcian Blue (Sigma-Aldrich, Cat#A5268).

### Non-steroidal anti-inflammatory drugs (NSAIDs)

NSAIDs (Ibuprofen (Sigma; Cat#I4883), Ketorolac tris salt (Sigma; Cat#K1136), Indomethacin (Sigma; Cat#I7378)) and the selective COX2 inhibitor Celecoxib (Sigma; Cat# PZ0008) were purchased, stored at RT, and diluted according to manufacturer’s specifications. Concentrations were tested according to previous studies based on pharmacokinetics of plasma levels corresponding to typical and maximum intake. NSAIDs were administered to cells *in vitro* at peak plasma levels corresponding to therapeutic levels reported in pharmacokinetic analyses and as indicated in the figures (19–21).

### Lentiviral overexpression

A lentivirus plasmid to overexpress COX2 with a dTomato tag was constructed using Gibson cloning of pHIV-dTomato (Addgene cat# 21374) and PTGS2 (Origene Cat#SC128243). HEK-293T cells were transfected using calcium phosphate transfection with VSV-G (addgene Cat #8454), psPAX2 (addgene Cat #12260), and either ZsGreen (addgene Cat#18121) or PTGS2 dTomato. Lentiviral particles were concentrated using Lenti-X concentrator (Takara, Cat#631232) and then immediately used to transduce hSSCs plated one hour prior at 80% confluency at a dilution of 1:100 with 1:1000 polybrene. Confluent cells were FACS sorted by fluorescence for subsequent expansion and differentiation.

### Subcutaneous transplantation of human skeletal stem cells

Freshly sorted patient derived hSSCs were sorted and expanded to confluency. 2x10^6^ cells were mixed with 5 ul of Matrigel and seeded on 20 mg anorganic cancellous bone graft granulat (InterOss^®^, 0.25-1mm) at 4°C. The solution was transferred to a round-bottom 96-well plate well and allowed to solidify for 5 minutes at room temperature. The gelatinized cell mixture was then transplanted subcutaneously in the dorsum of NSG mice. PBS or Indomethacin was administered at 2 mg/kg for the first 7 days after cell transplant. Grafts were excised and analyzed 4- and 8-weeks later.

### Micro-CT analysis of grafts

Grafts were dissected from mice and fixed in 2% PFA overnight. The next day grafts were transferred to tubes containing sterile water and scanned using a Bruker Skyscan 1276 (Bruker Preclinical Imaging) with a source voltage of 85 kV, a source current of 200 µA, a filter setting of AI 1 mm, and pixel size of 12 microns at 2016 x 1344. Reconstructed samples were analyzed using CT Analyser (CTan) v1.17.7.2 and CTvox v3.3.0 software (Bruker). Sections spanning the size of the graft were selected and upper (255) and lower (60) grey threshold were set. The total mineralized volume was measured for each graft assuming equal starting amounts of anorganic cancellous bone graft granulat.

### Transcriptomic analysis

Transcriptomic analysis was performed on highly purified, double-sorted mSSCs, mBCSPs, hSSC, hOP, and hCP populations either directly sorted into TRIzol LS (Invitrogen, Cat#10296028) or expanded and differentiated towards the osteogenic lineage for 14 days before collection in TRizol. RNA was isolated with RNeasy Micro Kit (QIAGEN, Cat#74004) as per manufacturer’s instructions. For microarray analysis RNA was twice amplified with an Arcturus RiboAmp PLUS Kit (Applied Biosystems, Cat#KIT0521). Amplified cRNA was streptavidin-labeled, fragmented, and hybridized to Affymetrix arrays HG-U133+ (for human genome; Applied Biosystems, Cat#901569). Arrays were scanned with a Gene Chip Scanner 3000 (Affymetrix) running GCOS 1.1.1 software. Raw microarray data was submitted to Gene Expression Commons (https://gexc.riken.jp/models/2551 and https://gexc.riken.jp/models/2552). On this platform data is normalized by computing against the Common Reference, which is comprised of a large number of array (mouse 11,939 and human 25,229) experiments deposited to the National Institutes of Health Gene Expression Omnibus (NIH GEO) database. GEXC assigns a threshold value to each probeset using the StepMiner algorithm and calculates a percentile value between -100% (inactive) and +100% (active) for each available gene, allowing comparison of human gene expression on a normalized, continuous scale. From there, heatmaps were generated showing fold change in gene expression of Cyclooxygenase mRNAs. For quantitative PCR experiments the following primers were used for timecourse experiments with hSSCs: *COX-1* (PTGS1; NM_000962.4), F-GATGAGCAGCTTTTCCAGACGAC, R-AACTGGACACCGAACAGCAGCT; *COX-2* (PTGS2; NM_000963), F- CGGTGAAACTCTGGCTAGACAG, R-GCAAACCGTAGATGCTCAGGGA.

### Histochemistry

Cryo-sections were stained using Movat’s Pentachrome or hematoxylin and eosin (H&E). Adjacent sections were used for immunofluorescence (IF) with primary antibodies mouse anti-human Human Nuclear Antigen (HNA; Abcam, Cat#ab191181) and rabbit anti-human Osteocalcin (OC; Abcam, Cat#ab93876) at 1:200 dilutions. The secondary antibodies goat anti-mouse AF-488 (Abcam, Cat#ab150117) and donkey anti-rabbit AF-647 (Abcam, Cat# ab150075) were added at 1:500 dilutions and sections counterstained with DAPI. Fluorescence microscopy (Leica TCS Sp8) was used to capture images.

### Immunocytochemistry

For immunocytochemistry, fixated cells in well plates were permeabilized with 0.1% Triton X-100 solution and blocked with 3% BSA in PBS. After incubation with primary antibody for Cox-2 [ThermoFisher, Cat#12375-1-AP]) overnight at 4°C, secondary antibody was applied for 30 min at room temperature. For nuclear staining specimen were treated with DAPI (BioLegend, Cat# 422801). Fluorescence Quantification of Cox-2 expression in cultured hSSCs was measured by Corrected Total Cell Fluorescence (CTCF) and calculated using ImageJ for cells of five independent donors and for each time point. Each CTCF value is the average of five cells that is the integrated density minus the area of the selected cells multiplied by the mean fluorescence of the background readings.

### Statistics

Data are presented as mean + standard error of the mean (SEM). Experiments were conducted at least in duplicate as indicated in the figure legends. Statistical analysis between two experimental groups was determined using two-tailed, unpaired Student’s t-test. Normality was assessed by Shapiro-Wilk test and corrected if failed by using Mann-Whitney test. If unequal variances (F-test) were detected the t-test was adjusted with Welch’s correction. For comparison of more than two groups one-way ANOVA analysis was used with Tuckey’s posthoc test. P-values were considered significant if p < 0.05. Statistical analyses were performed using GraphPad Prism 9 (GraphPad).

## Results

**Figure 1:**
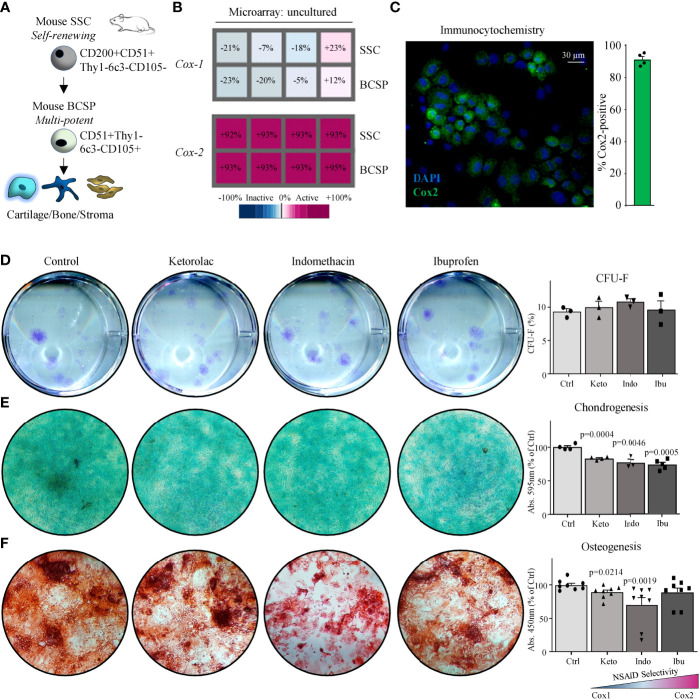
Mouse skeletal stem cells depend on Cox2 for functional osteochondrogenic differentiation. **(A)** The mouse skeletal stem cell (SSC) lineage tree as defined by surface marker expression profiles with the SSC at the apex and the downstream bone cartilage stroma progenitor (BCSP) which gives rise to committed bone, cartilage, and stroma progenitor cells. **(B)** Microarray analysis showing *Cox-1* and *Cox-2* expression of freshly purified SSCs and BCSPs from fracture calluses of four different mice. **(C)** Representative immunocytochemistry (ICC) staining of Cox2 of fracture-derived SSCs expanded in culture for five days. **(D)** Representative images of colony-forming unit assays of fracture-derived SSCs stained with Crystal Violet expanded in the absence or presence of NSAIDs (left; Ketorolac 0.3 µg/ml, Ibuprofen 3 µg/ml, or Indomethacin 0.3 µg/ml) and quantification thereof (right). Replicates from n=3 mice. **(E)** Representative images of chondrogenesis assays of fracture derived SSCs stained with Alican Blue differentiated in the absence or presence of NSAIDs (left) and quantification thereof (right). Replicates from n=4 mice. **(F)** Representative images of osteogenesis assays of fracture derived SSCs stained with Alizarin Red S differentiated in the absence or presence of NSAIDs (left) and quantification thereof (right). Replicates from n=4 mice. All data shown as mean + standard error of mean (SEM). Results from at least two independent experiments. Statistical testing versus control group by unpaired Student’s t-test with Welch’s correction for unequal variances and Mann-Whitney test for non-normality where necessary.

Microarray data of freshly purified skeletal lineage cell populations from day-10 mid-diaphyseal femoral fractures (**Figures S1A, B**) demonstrated that *Cox-2* but not *Cox-1* mRNA was abundantly expressed in freshly isolated mouse SSCs (CD45-Ter119-Tie2-Thy1-6c3-CD51+CD105-CD200+) and BCSPs (CD45-Ter119-Tie2-Thy1-6c3-CD51+CD105+) (**Figures 1A, B**). Additionally, primary mouse SSCs (mSSCs) cultured *in vitro* expressed high levels of COX2 (**Figure 1C**). When we seeded freshly sorted fracture mSSCs at clonal density and continuously treated expansion cultures with the common NSAIDs Ketorolac (Keto), Indomethacin (Indo), or Ibuprofen (Ibu) we did not observe any changes to the fibroblast colony forming unit (CFU-F) ability as well as the size of the colonies compared to controls (**Figures 1D** and **S1C**). This suggested that proliferation of mSSCs was most likely not affected by NSAID treatment. Next, we examined whether NSAIDs inhibited chondrogenic and osteogenic differentiation capacity of mSSCs *in vitro*. All three NSAIDs tested significantly inhibited chondrogenic differentiation in mSSC compared to controls as determined by Alcian Blue staining quantification (**Figure 1E**). Similarly, *in vitro* osteogenic mineralization was strongly diminished in the presence of Ketorolac and Indomethacin (**Figure 1F**). These results extend the previously reported inhibitory effect of Cox2-inhibition on bone biology in rodents to the purified skeletal stem cell level, indicating a direct role in perturbing endochondral bone formation processes.

**Figure 2:**
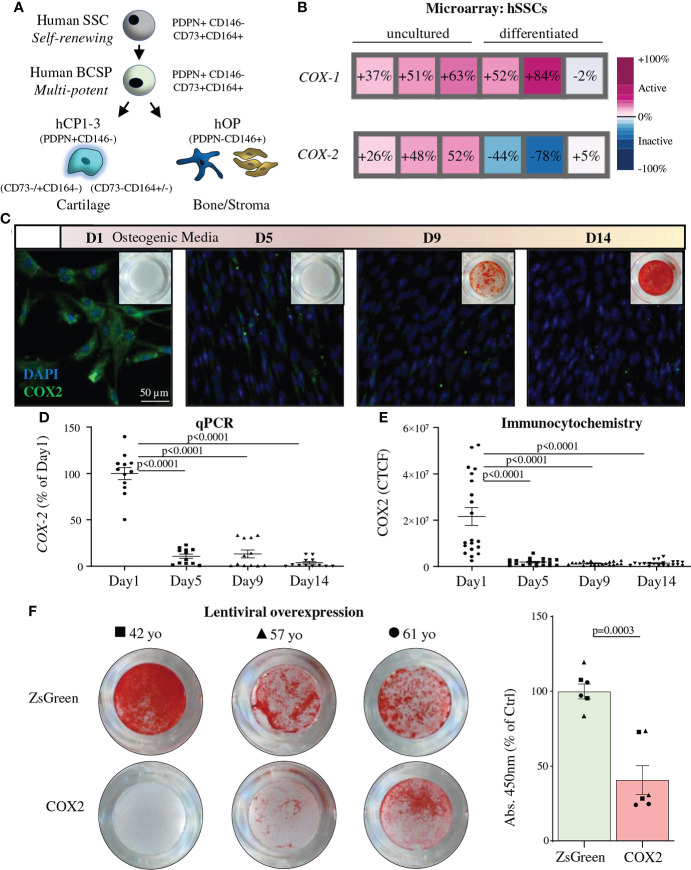
COX2 downregulation is necessary for osteogenic differentiation of human SSCs. **(A)** The human skeletal stem cell (hSSC) lineage tree as defined by surface marker expression profiles with the hSSC at the apex and the downstream bone cartilage stroma progenitor (hBCSP) which gives rise to committed bone (hOP) and cartilage (hCPs) progenitor cells. **(B)** Microarray analysis showing *COX-1* and *COX-2* expression of freshly purified (uncultured) hSSCs from human fracture callus tissue of three different patients as well as their expression after two-week osteogenic differentiation from hSSCs. **(C)** Representative IHC images of COX2 staining in freshly purified hSSCs at different timepoints during *in vitro* osteogenesis. **(D)** Related quantitative PCR of *COX-2* expression in the same experiment. n=12 independent replicates of hSSCs from four patients performed in triplicates. **(E)** Quantification of COX2 ICC staining by CTCF (corrected total cell fluorescence). n=20 independent replicates of hSSCs from two patients (n=10 each). Statistical testing between timepoints by one-way ANOVA with Tukey’s posthoc test. **(F)** Alizarin Red staining and quantification of hSSCs (with patient age; yo: years old) of lentivirally overexpressed COX2 or ZsGreen controls during osteogenic differentiation. n=6 independent replicates of hSSCs from three patients performed in duplicate. Statistical testing by unpaired Student’s t-test. All data shown as mean ± SEM. All results from at least two independent experiments.

Next, we examined whether COX enzymes played a functional role in human SSCs (hSSCs). We purified human hSSCs (CD45-CD235-CD31-TIE2-CD146-PDPN+CD164+CD73+), as well as their downstream osteoprogenitors (hOPs) and chondroprogenitors (hCPs), from acute fractures at the time of surgical open reduction and internal fixation (**Figure 2A** and **Figures S2A, B**). Transcriptomic analysis revealed that *COX-1* mRNA was stably expressed in purified uncultured and differentiating patient-derived hSSCs (**Figure 2B** and **Figure S2C**). *COX-2* expression, on the other hand, was high in freshly isolated hSSCs but rapidly lost gene and protein expression upon early commitment towards the osteogenic lineage as shown by qPCR and immunocytochemistry time-course analyses (**Figures 2B–E**). In line with this observation, *COX-2* expression was not detectable in freshly isolated committed osteoprogenitors (hOPs; CD45-CD235-CD31-TIE2-CD146+PDPN-) (**Figures S2D, E**). Interestingly, early chondrocyte progenitor cells (hCPs) maintained high levels of *COX-1* and *COX-2* expression. To test the influence of the presence of *COX-2* in hSSCs during osteogenic differentiation we lentivirally overexpressed primary hSSCs with a *COX-2* construct and induced osteogenesis. Compared to GFP-transduced cells, hSSCs from three different patients showed strongly diminished *in vitro* mineralization when *COX-2* was continuously expressed (**Figure 2F**). Taken together, this data indicates that primary bona fide hSSCs express *COX-2* in an undifferentiated state but, in contrast to mSSCs, might depend on its downregulation for osteogenic differentiation.

**Figure 3:**
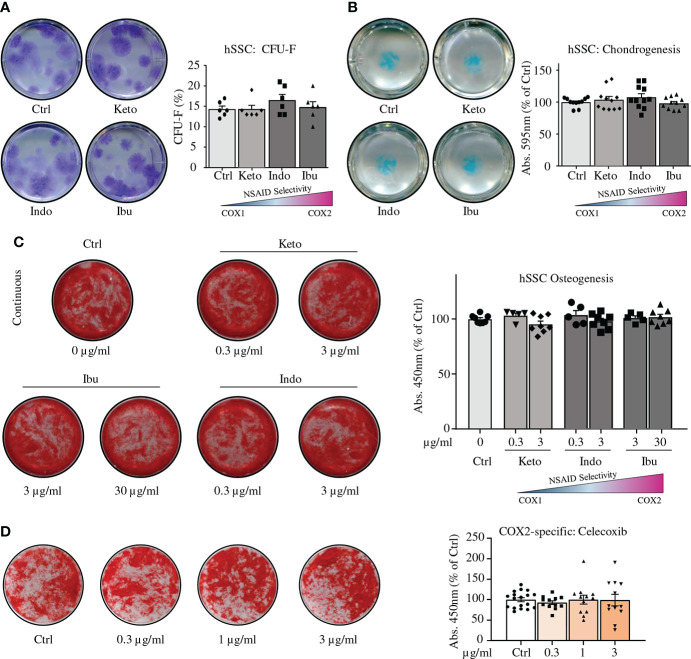
NSAIDs do not alter *in vitro* functionality of human SSCs. **(A)** Representative images of CFU-F assays of fracture derived hSSCs stained with Crystal Violet expanded in the absence or presence of NSAIDs (left; Ketorolac 3 µg/ml, Ibuprofen 30 µg/ml, or Indomethacin 3 µg/ml) and quantification thereof (right). n=6 independent replicates from hSSCs of two patients performed in triplicate. **(B)** Representative images of chondrogenesis assays of fracture derived hSSCs stained with Alican Blue differentiated in the absence or presence of NSAIDs (left) and quantification thereof (right). n=11 independent replicates from hSSCs of five patients performed at least in duplicate. **(C)** Continuous NSAID treatment of osteogenic differentiation assays from hSSCs. n=8 independent replicates from hSSCs of four patients performed in duplicate. **(D)** Osteogenic differentiation of hSSCs in the presence of COX2-specific inhibitor Celecoxib. n=12-18 independent replicates from hSSCs of six patients performed at least in duplicates. All data shown as mean ± standard error of mean (SEM). All results from at least two independent experiments. Statistical testing versus control group by unpaired Student’s t-test with Welch’s correction for unequal variances where necessary.

Next, we surveyed reported peak plasma levels of common NSAIDs in human patients and treated colony-forming unit fibroblast assays with the average of these concentrations (**Figure S3A**) (19–21). Ketorolac (3 µg/ml), Indomethacin (3 µg/ml), or Ibuprofen (30 µg/ml) treatment did not alter clonogenicity of hSSCs compared to controls (**Figure 3A**). In contrast to mSSCs, chondrogenesis of hSSCs was also unaffected in the presence of these NSAIDs (**Figure 3B**). Using *in vitro* bone-forming assays we tested low and peak plasma concentration levels of NSAID and treated hSSC cultures either short-term (first three days) or continuously (throughout differentiation) with NSAIDs. Regardless of NSAID supplementation and duration of treatment, there was no effect on osteogenic potential of hSSCs (**Figure 3C** and **Figures S3B, C**). Importantly, when we assayed the effect of the commonly used selective COX2-inhibitor Celecoxib at varying concentration, we could not observe any effect on osteogenic differentiation either (**Figure 3D**). Since NSAIDs could act downstream of the stem cell level or on a putative distinct SSC lineage, we also asked if short-term or continuous NSAID administration differentially affected osteogenic differentiation of CD146-positive osteoprogenitors (hOPs), previously described as a key source of bone formation in humans (22). We found that neither low nor high doses of NSAIDs added during differentiation, short-term or continuously, inhibited bone mineralization in hOPs (**Figures S3D-F**). This suggested a species-specific effect of COX-inhibition on bone-forming cell types between mice and humans.

**Figure 4:**
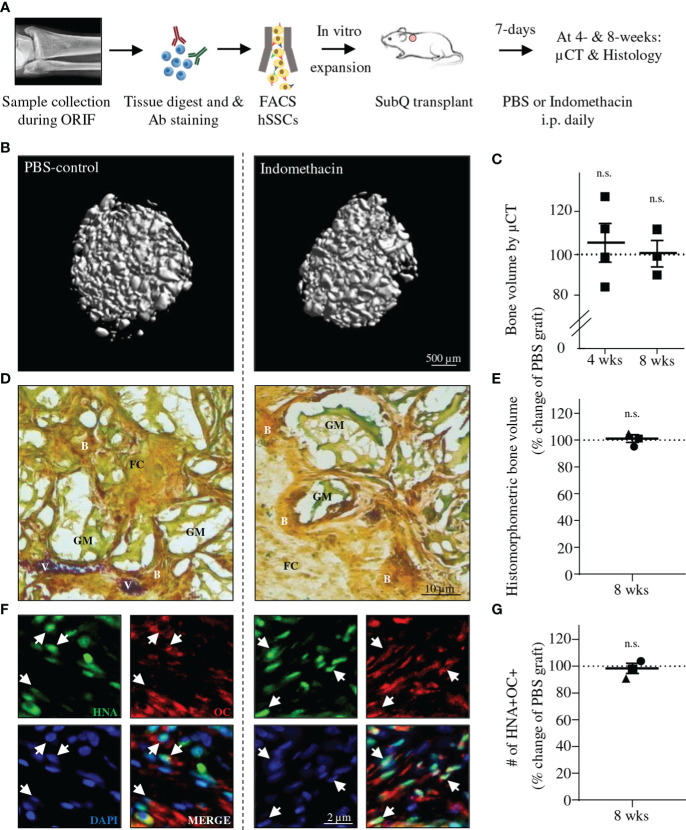
Indomethacin does not interfere with *in vivo* ossicle formation of human SSCs. **(A)** Experimental schematic for *in vivo* grafting of hSSCs and treatment of mice with Indomethacin. **(B)** Three-dimensional microCT reconstruction of mineralized graft tissue. **(C)** Quantification of mineralized graft tissues (total bone volume) at 4- (n=4) and 8-weeks (wks; n=3) post transplant between PBS and Indomethacin treated mice. Results from seven experiments with hSSCs from distinct patients. **(D)** Representative Movat Pentachrome staining of sectioned grafts (B, Bone; FC, Fibrocartilage; V, Blood vessel; GM, Graft material). **(E)** Histomorphometric quantification of graft bone volume (each data point represents average of three non-adjacent sections per patient hSSC-derived graft). **(F)** Immunohistochemistry showing graft derived osteogenic cells are of human origin. Human Nuclear Antigen (HNA; green), Osteocalcin (OCN; red), DAPI (blue). **(G)** Quantification of OCN-expressing cells of human origin based on IHC. Data shown as mean + SEM. Statistical testing by paired Student’s t-test (n.s., not significant).

Lastly, we sought to explore if exposure to NSAIDs affects *de novo* bone formation reflective of the fracture healing process *in vivo*. SSCs are able to recreate skeletal structures, if transplanted as purified single cell solution to ectopic sites, provided access to vascular ingress (13, 14, 23). Thus, we transplanted primary patient-derived hSSCs subcutaneously into immune-incompetent NSG mice that were treated intraperitoneally with Indomethacin or PBS as control daily for one week (**Figure 4A**). We reasoned that the lack of adaptive immunity in NSG mice would be well suited to assess a direct effect of NSAIDs on the hSSC function. As expected, patient hSSCs generated grafts complete with bone tissue at least in part forming through a cartilage intermediate (**Figures 4B–E** and **Figures S4A–C**). At four and eight weeks, µCT analysis showed no differences in mineralization between hSSC-derived grafts from seven independent patients either transplanted into mice receiving NSAID doses or to patient-matched PBS controls (**Figure 4C**). Histomorphometric quantification of bone tissue in grafts confirmed these results. Importantly, immunostaining revealed that osteocalcin-expressing cells in grafts were of human origin and did not differ in frequency between groups (**Figures 4F, G**). In summary, osteochondrogenic differentiation of fracture-derived hSSC lineage populations is facilitated in the absence of COX2, providing a rationale for the discrepancy observed between animal experiments and human studies.

## Discussion

The effects of NSAIDs on osteogenic stem cell differentiation and fracture healing remain controversial and appear to vary with investigated species and cell type. Here, we compared prospectively isolated, highly purified, homologous populations of skeletal stem cells from mice and humans and report that COX enzymes may be dispensable for chondrogenic and osteogenic differentiation in fracture-derived hSSCs but not mSSCs.

In mice we found that NSAIDs repressed chondrogenic and osteogenic differentiation of mSSCs from fractures. In general, NSAIDs appear to inhibit murine fracture healing based on previous evidence. Perhaps most convincingly, genetic *Cox-2* null mice demonstrate bone healing defects (5). From the perspective of bone marrow stromal cells, it has previously been reported that NSAIDs are inhibitory at the osteogenic differentiation level, although in a non-cell autonomous model (10). Although NSAIDs had no effect on serum markers of fracture healing or biomechanical properties of fracture callus in rat fractures (24), there is also evidence that in rat long bone fractures, prolonged NSAID administration inhibits BMSC differentiation and fracture callus formation (25). We have extended these observations by demonstrating an inhibitory effect of NSAIDs on osteochondrogenic differentiation in the highly purified and characterized mSSC, a bona fide skeletal stem cell, that has been shown to play an essential part in fracture healing (14–17, 26, 27). However, the limitations translating genetically homogenous mouse models to complex multi-factorial disease processes are well-documented (28, 29). Subsequently, we have also studied the homologous human cell population, the hSSC, and made distinct observations (13, 16).

We found no effect of NSAIDs on osteogenic and chondrogenic differentiation of hSSCs, which is not fully concordant with many previous human studies but is consistent with the strongest available clinical evidence. A previous analysis of human bone marrow stromal cells did find a specific inhibitory effect of multiple NSAIDs on chondrogenesis but not osteogenic differentiation (9), while another study demonstrated an inhibitory effect of NSAIDs on osteogenesis (30). These conflicting results might have several explanations, including the fact that cells were derived from uninjured tissue through bone marrow aspirates as well as pro-longed selection and growth in culture. The present study used flow cytometrically purified defined skeletal progenitor cell types with minimal *in vitro* expansion. Our earlier work could show that fracture-derived SSCs behave differently than their steady-state counterparts (27) and that selection of heterogenous cell populations by plastic adherence leads to variations in experimental readout (11, 12, 31). Strikingly, in this study we also observed a lack of effect of NSAIDs on differentiation of lineage restricted osteoprogenitors (hOPs; CD146-positive), which contain a previously described populations of perivascular bone marrow stromal cells with stem cell-like features (22). It is feasible to assume that by initiating differentiation experiments at the hSSC level with cells undergoing maturation through more committed lineage progenitor stages before terminal osteochondral differentiation, our findings of a lack of effect of NSAIDs on experimental outcome can be extrapolated to human BMSCs, which contain a heterogeneous mixture of stem and progenitor cell types. While COX1/2 are expressed at time of isolation of hSSCs from fracture sites, COX2 expression becomes attenuated during differentiation, suggesting an alternate role, if any for this enzyme at sites of skeletal injury. We also observed that lentiviral overexpression of COX2 in hSSCs actually prevented *in vitro* mineralization. This could be a consequence of superphysiological COX2 levels. Moving forward this could be mitigated by using more specific transcriptional control with other genetic models. We will also have to test if the same results are obtained when a catalytically inactive COX2 variant is used as a control.

Our work might have not covered cell types highly enriched for “MSC”-like cells that have been shown to modulate local and systemic inflammatory responses (32), and may do so at sites of fracture as well. Future studies will have a closer look at NSAID effects on angiogenesis and immune cells during early fracture healing in humans. However, our *in vivo* results suggest that even in a monocyte/macrophage enriched environment, as present in NSG mice, which have been implicated in NSAID mediated suppression of osteogenesis, bone formation from SSCs is not impaired in the presence of NSAID drugs (10).

In the clinical setting, there remains a lack of convincing evidence, but not controversy, surrounding the use of NSAIDs in fracture healing (5–7, 33). A recent meta-analysis concluded that association of nonunion with NSAID use was predominantly found in studies with insufficient cohort sizes, unclear definition of outcomes and even fraud allegations, stating that there were a dearth of high quality studies in fracture literature (8). Another review cited lack of strong evidence against NSAID use in fracture healing (34).

In conclusion, there is great interest in safe post-operative analgesia, given the current opioid crisis, especially during fracture care in orthopaedic surgery. Currently, much of our understanding of the role of NSAIDs in fracture healing comes from rodent models. Here, we demonstrate that NSAIDs have disparate and species-specific effects on osteochondrogenic differentiation of homologous populations of murine and human fracture-derived SSCs, which are prospectively isolated and a highly purified cell population. In contradistinction to the mSSC, the hSSC is unaffected by NSAID administration. COX enzyme-specific mechanisms in SSCs likely evolved to synchronize priming of SSC-dependent regenerative responses with recruitment of inflammatory cell types that may also facilitate other aspects of the regenerative process. COX1 might be differentially regulated at the stem cell level in mice while, for instance, recent findings have also shown that there are differences between humans and mice in their regulation of COX2 expression (18). Finally, the expression of COX1 versus COX2 in human versus mouse SSCs is a species-specific regulatory switch that might serve to maintain stem cell identity rather than promoting differentiation. Thus, caution should be used in extrapolating mechanistic data from experimental animal models to clinical practice. Our data provides evidence from a mechanistic perspective that NSAID does not appear to impair human skeletogenic stem and progenitor cells and contributes to the hypothesis that NSAID use might be safe after fractures in humans in some contexts. Additionally, hSSCs isolated from acute human fractures provide a model with which to study how common medications may influence fracture healing.

## Data availability statement

The microarray data presented in the study haven been deposited in public repositories. All raw and processed data are available from the NCBI GEO database with accession number GSE211910. Data browsing is also available in the Gene Expression Commons depository (GEXC) at https://gexc.riken.jp/models/2551 and https://gexc.riken.jp/models/2552.

## Ethics statement

The studies involving human participants were reviewed and approved by Stanford IRB. Written informed consent for participation was not required for this study in accordance with the national legislation and the institutional requirements. The animal study was reviewed and approved by Stanford’s Administrative Panel on Laboratory Animal Care.

## Author contributions

LG, TA, and CC conceived and designed the study. MGB, LG, TA, HS, and CC conducted experiments, analyzed and interpreted data, and wrote the manuscript. MH, HC, NR, MJB, JB, and MG collected samples and supported analysis. CC supervised the research. All authors contributed to the article and approved the submitted version.

## Funding

This work was supported by NIH/NIA K99 R00 AG049958-01A1, 1S10OD028493-01A1, Heritage Medical Foundation, American Federation for Aging Research (AFAR)/Arthritis National Research Foundation (ANRF), Wu Tsai Human Performance Alliance and an endowment from the DiGenova Family to CC, the German Research Foundation (DFG-Fellowship) 399915929 and NIH/NIA 1K99AG066963 to TA. This work was also supported by CIRM Bridges 3.0 #EDUC2-12620 to MGB and the Young Investigator Research Award from the AO North America to LG. Additional support came from NIH S10 RR02933801 to Stanford University Stem Cell FACS core and NIH S10 1S10OD02349701 to Stanford University Clark Imaging Center (PI: Timothy Doyle).

## Acknowledgments

We thank P. Lovelace, S. Weber, C. Carswell-Crumpton for FACS support. We also thank M.R. Eckart and the Stanford Gene Expression Facility (PAN Facility) as well as Stanford Human Immune Monitoring Center (HIMC) for technical support, assistance, and/or advice on this project. We also thank Dr. Pamela Robey for advice on bone grafts for in vivo experiments.

## Conflict of interest

The authors declare that the research was conducted in the absence of any commercial or financial relationships that could be construed as a potential conflict of interest.

## Publisher’s note

All claims expressed in this article are solely those of the authors and do not necessarily represent those of their affiliated organizations, or those of the publisher, the editors and the reviewers. Any product that may be evaluated in this article, or claim that may be made by its manufacturer, is not guaranteed or endorsed by the publisher.
