# Supplementary material for: Cross-species comparisons reveal resistance of human skeletal stem cells to inhibition by non-steroidal anti-inflammatory drugs
Source: Front Endocrinol (Lausanne). 2022 Aug 25;13:924927. doi: 10.3389/fendo.2022.924927 (PMC9454294; doi:10.3389/fendo.2022.924927)
Supplement: Supplementary file 1 [file Image_1.pdf]

Supplemental Figure 1

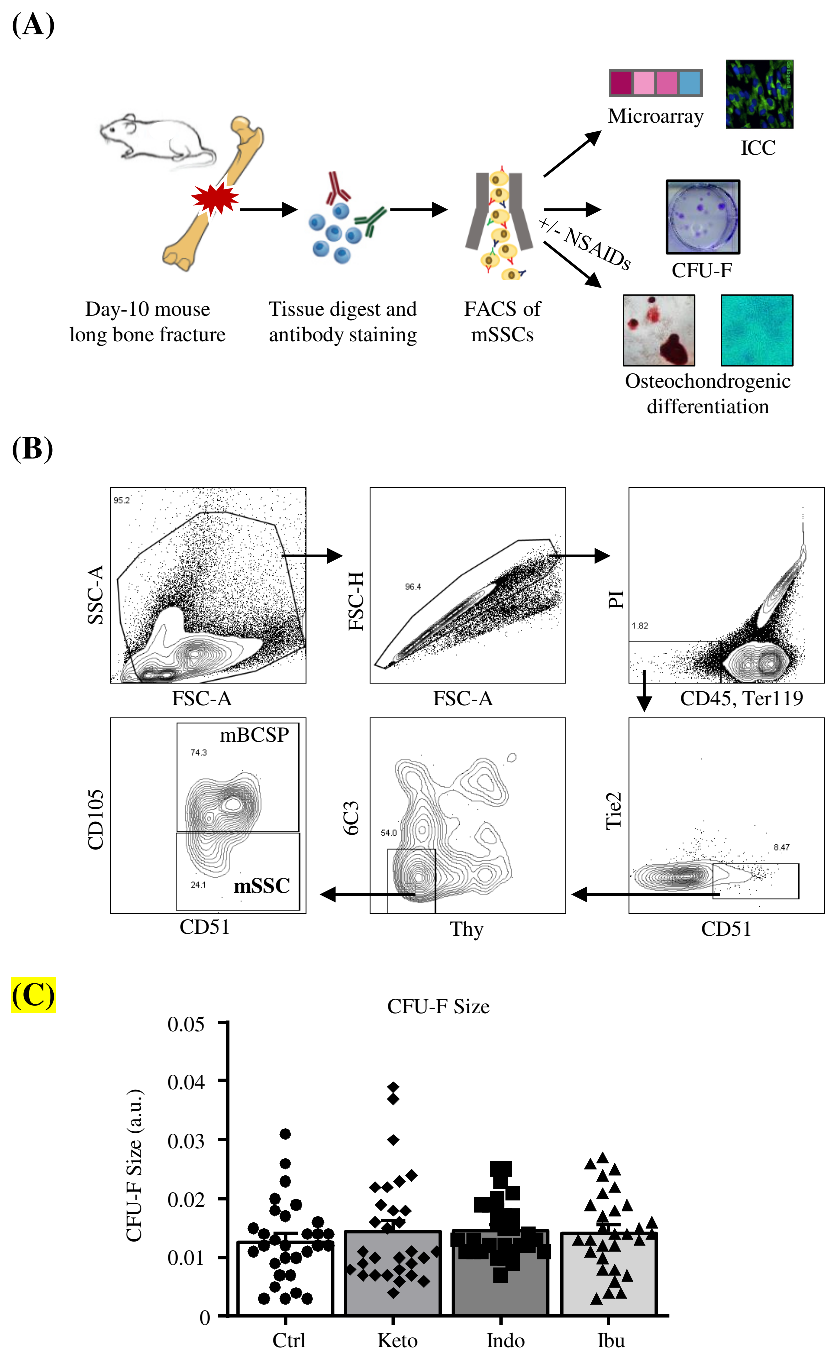

Supplemental Figure 1: Probing in vitro NSAID effects on mouse SSC function. **(A)** Schematic of tissue processing, SSC isolation, and performed in vitro assays. **(B)** Representative gating strategy for the isolation of fracture derived human SSCs. **(C)** Quantification of CFU-F size (a.u.: arbitrary unit; n=30 per group from three independent replicates). Data shown as mean + standard error of mean (SEM). Statistical testing versus control group by unpaired Student's t-test.
