# Supplementary material for: Cross-species comparisons reveal resistance of human skeletal stem cells to inhibition by non-steroidal anti-inflammatory drugs
Source: Front Endocrinol (Lausanne). 2022 Aug 25;13:924927. doi: 10.3389/fendo.2022.924927 (PMC9454294; doi:10.3389/fendo.2022.924927)
Supplement: Supplementary file 2 [file Image_2.pdf]

Supplemental Figure 2

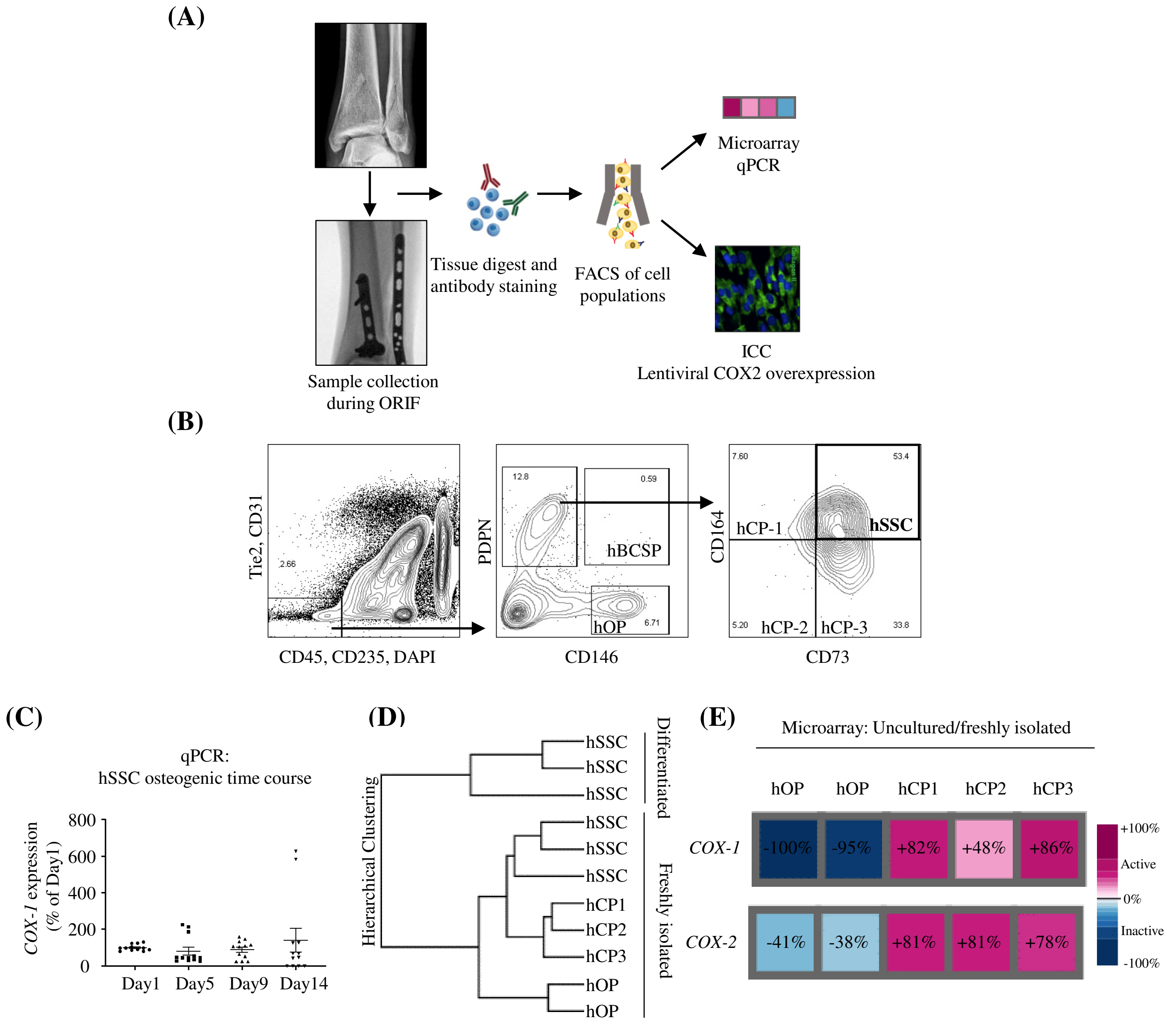

Supplemental Figure 2: Differential expression of COX enzyme in human skeletal lineage subpopulations. (A) Schematic of cell isolation and performed in vitro assays on human SSCs. **(B)** FACS gating strategy for the prospective isolation of hSSCs, hOPs, and hCPs. **(C)** Quantitative PCR of *Cox-1* expression in the time course of hSSC in vitro osteogenesis. n=12 individual replicates of hSSCs from four patients performed in triplicates. Statistical testing between timepoints by one-way ANOVA with Tukey's posthoc test. **(D)** Hierarchical clustering of human skeletal lineage populations freshly isolated and osteogenically differentiated based on their overall transcriptomic profile by microarray. **(E)** *COX-1* and *COX-2* gene expression as determined by microarray in freshly isolated hOP and hCP populations. n=2 patients. All data shown as mean ± SEM. Results from at least two independent experiments.
