# Supplementary material for: Cross-species comparisons reveal resistance of human skeletal stem cells to inhibition by non-steroidal anti-inflammatory drugs
Source: Front Endocrinol (Lausanne). 2022 Aug 25;13:924927. doi: 10.3389/fendo.2022.924927 (PMC9454294; doi:10.3389/fendo.2022.924927)
Supplement: Supplementary file 3 [file Image_3.pdf]

Supplemental Figure 3

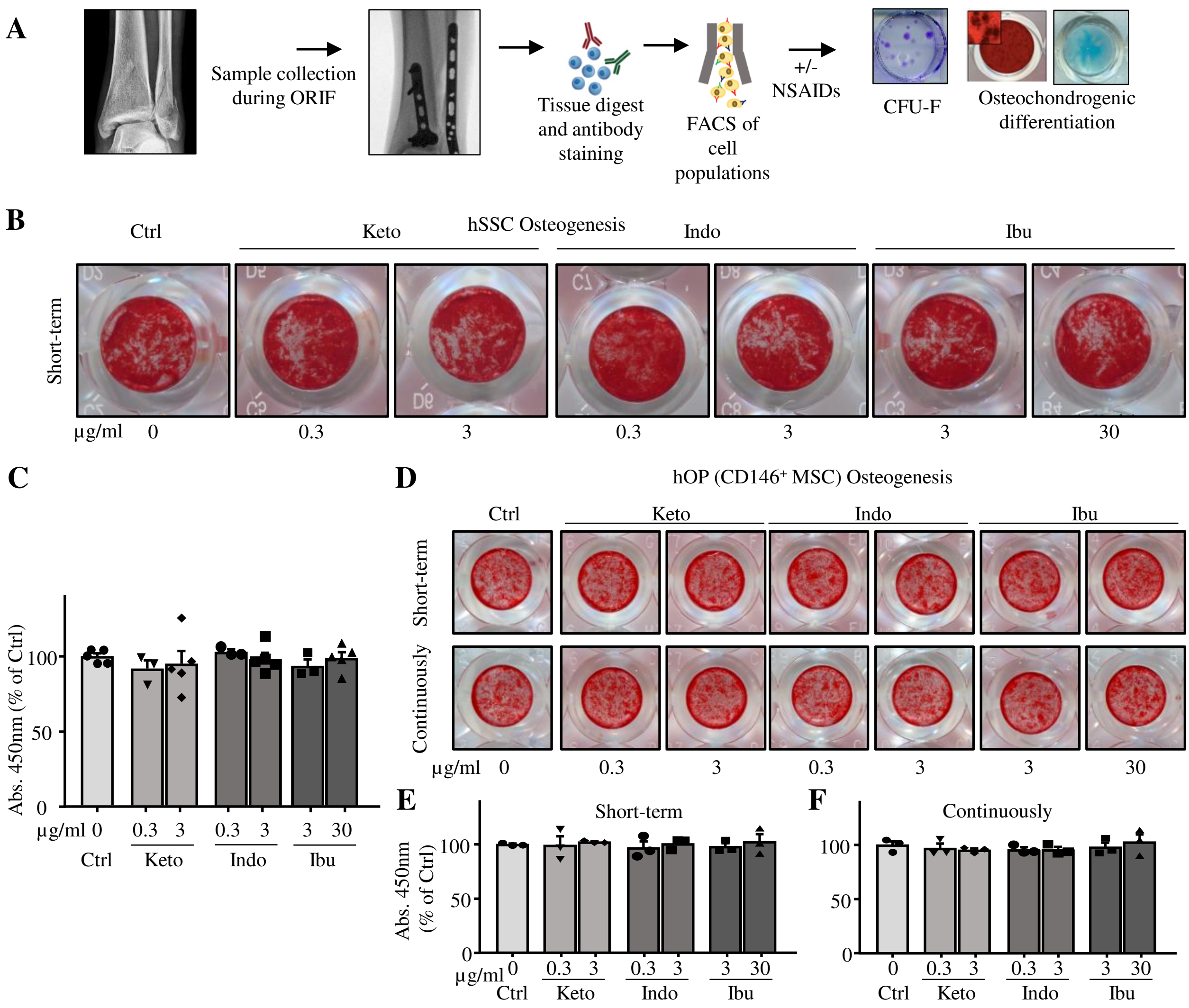

Supplemental Figure 3: NSAIDs do not alter in vitro osteogenesis of human osteoprogenitors (hOPs). (A) Schematic of isolation of cells from fracture samples and in vitro assays performed. **(B)** Representative images of osteogenesis assays of fracture derived hSSCs stained with Alizarin Red S differentiated in the absence or presence of NSAIDs for the first three days (short-term) of osteogenesis. **(C)** Quantification of short-term NSAID-treated hSSC osteogenesis. n=3-6 individual replicates of donor hSSCs. **(D)** Representative images of osteogenesis assays of fracture derived hOPs stained with Alizarin Red S differentiated in the absence or presence of NSAIDs for short-term or the whole course (continuous) of osteogenesis. **(E)** Quantification of short-term NSAID-treated hOP osteogenesis. n=3 individual replicates of donor hSSCs. **(F)** Quantification of continuously NSAID-treated hOP osteogenesis. n=3 individual replicates of donor hSSCs. All data shown as mean + SEM. Results from at least two independent experiments. Statistical testing versus control group by unpaired Student's t-test with Welch's correction for unequal variances where necessary.
