# Supplementary material for: Cross-species comparisons reveal resistance of human skeletal stem cells to inhibition by non-steroidal anti-inflammatory drugs
Source: Front Endocrinol (Lausanne). 2022 Aug 25;13:924927. doi: 10.3389/fendo.2022.924927 (PMC9454294; doi:10.3389/fendo.2022.924927)
Supplement: Supplementary file 4 [file Image_4.pdf]

Supplemental Figure 4

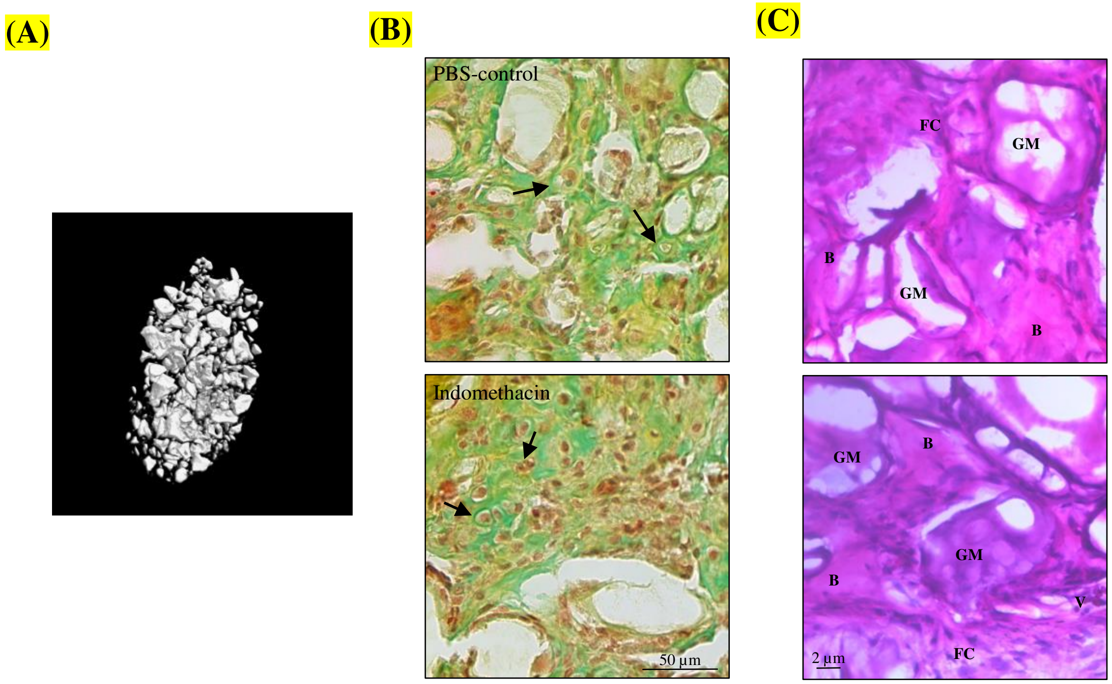

Supplemental Figure 4: In vivo human SSC bone formation proceeds at least in part through a cartilage intermediate. (A) Micro-CT image of 8-week-old control graft showing lack of mineralization between InterOss graft material. **(B)** Movat's Pentachrome staining of 4-week human SSC-derived grafts staining cartilage in blue. Arrows pointing at phenotypic chondrocyte. **(C)** Representative higher magnification H&E staining of sectioned 8-week grafts (B: Bone; FC: Fibrocartilage; V: Blood vessel; GM: Graft material).
